# Effects of tDCS and intensive behavioral therapy in a longevous individual with low education and subacute aphasia

**DOI:** 10.1590/2317-1782/e20240304en

**Published:** 2026-01-30

**Authors:** Natália Callegaro Costa, Rafaela Rossini Peters, Pamela Lemes Rocha, Karina Carlesso Pagliarin

**Affiliations:** 1 Departamento de Fonoaudiologia, Universidade Federal de Santa Maria – UFSM - Santa Maria (RS), Brasil.

**Keywords:** Transcranial Direct Current Stimulation, Stroke, Speech Therapy, Aphasia, Speech

## Abstract

The aim of this study was to examine the effects of tDCS during intensive word-finding therapy on subacute aphasia after stroke. A single-case study with an 86-year-old man with four years of formal education, diagnosed with an ischemic stroke in the left hemisphere, resulting in non-fluent aphasia of the mixed type, accompanied by acquired apraxia of speech. For this purpose, the Montreal-Toulouse Language Assessment Battery - Brief Version was administered at five different time points: (A1) one week before the start of treatment; (A2) after the first session; (A3) after five sessions; (A4) after ten sessions and; (A5) after 30 days without intervention. The Naming and Visual Recognition Test was also administered at points A1, A4 and A5. After the intervention, improvements were noted in word naming and verbal word comprehension. There was a reduction in linguistic errors such as paraphasias. These results support the combination of tDCS and behavioral training.

## INTRODUCTION

Aphasia is an acquired disorder of language components that can affect the comprehension and/or production of language, oral, written, or both. It can affect access to vocabulary, syntactic organization, and the encoding and decoding of messages^(1)^. Aphasia primarily affects stroke survivors, with approximately one-third of them experiencing this disorder^(1)^.

It is thus crucial to intervene as early as possible in these situations to achieve satisfactory results^(2)^. Behavioral treatments for language recovery are widely used and effective, with most interventions focusing on word retrieval due to the high prevalence of anomia in individuals with aphasia^(3)^.

Currently, transcranial direct current stimulation (tDCS) is being investigated as an aid in behavioral therapy for people with aphasia^(4)^. tDCS consists of a non-invasive cortical modulation technique. For this purpose, a continuous low-intensity electric current is applied to the scalp via two electrodes, which causes a change in the resting potential of the neuronal membrane^(5)^.

The potential benefit of tDCS in speech and language therapy after stroke has been described since 2008^(5)^. Positive results have been observed in some studies, such as the restoration of the balance of activity between the hemispheres^(6)^ and improvements in all aspects of communication, especially in naming tasks^(7)^.

Several studies have shown the applicability of tDCS in non-fluent aphasia^(8)^, including in chronic cases. However, few studies are conducting tDCS in subacute conditions. People with aphasia at this stage have higher recovery rates, making it possible to obtain an effective therapeutic window^(2)^.

In addition to the timing of the neurological injury, factors such as education, occupation, and age also have a direct influence on the rehabilitation process of people with aphasia^(9)^. In Brazil, the Brazilian Institute of Geography and Statistics (IBGE) estimated that in 2022, 16% of the elderly population is illiterate^(10)^. A lower level of education and a lower occupational status are associated with a higher severity of aphasia^(11)^. It is therefore important to consider sociodemographic aspects alongside the post-stroke period.

However, there are still only a few studies linking tDCS alone or in combination with behavioral word-finding therapy to speech and language disorders in patients with acute or subacute aphasia. Although the results are positive, they are still quite limited^(12)^. Therefore, it makes sense to investigate the effects of tDCS in patients with subacute aphasia, as the recovery rate is higher in these first months.

Therefore, this study aimed to examine the effects of tDCS during intensive word-finding therapy on subacute aphasia after stroke in a long-lived individual with low education. Based on the literature, this study hypothesized that tDCS in addition to intensive behavioral word-finding therapy would improve or reduce the signs associated with aphasia.

## CASE REPORT

This is an analytical, cross-sectional, speech therapy intervention study of the case study type. The study was conducted according to the guidelines and regulatory standards for research involving human subjects established in Resolution 466/12 of the National Health Council and duly approved by the Research Ethics Committee of a higher education institution under number 055246. The participant signed the Free and Informed Consent Form.

J.M., male, Brazilian Portuguese-speaking, right-handed, 86 years old, four years of schooling, diagnosed with a simple ischemic stroke in the left hemisphere. The participant had no history of psychiatric or neurological disorders other than stroke, no epilepsy, and no metal implants in any part of the body.

He began speech therapy 30 days after the stroke, receiving one-hour sessions once a week for five months with the hospital speech therapy team, as he had non-fluent aphasia of the mixedcondu aphasia type in addition to acquired apraxia of speech.

On the day of the stroke, a computed tomography scan of the head was performed using a 64-channel multislice machine. Axial helical tomographic slices of the skull parallel to the orbitomeatal line were obtained in series without requiring an intravenous contrast medium. The findings indicated prominent sulci between the cortical gyri, the Sylvian fissures, and the cerebellar sheets. Discrete diffuse hypodensity of the periventricular white matter and calcified atheromas in the carotid siphons were also noted.

After the first month post-stroke, the participant underwent a repeat examination. A comparative analysis with the previous examination was performed. In this case, hypodensity was found in the left parietal region affecting the white and gray matter.

After completing the hospital’s standard speech-language therapy, the participant began the experimental protocol analyzed in this study. This protocol was initiated six months post-stroke. During the intervention period, the participant did not receive any additional speech-language therapy to isolate the effects of the proposed intervention.

Before starting treatment with tDCS, the questionnaire on sociodemographic data and health conditions was completed^(13)^. This questionnaire is a survey of demographic data, cultural (reading and writing) and communicative habits (family and social relationships), manual dominance, and medical history (aspects of general, sensory, and neurological health).

### Instruments

The participant was assessed at five different time points: (1) one week before the start of treatment; (2) after the first session; (3) after five sessions; (4) after 10 sessions; and (5) after 30 days without intervention using the Brief Language and Aphasia Assessment Battery (ABLA)^(14)^. This assessment format was based on other scientific studies that applied tDCS in people with aphasia^(8)^.

The ABLA Battery consists of 10 tasks: directed interview, which assesses oral comprehension quantitatively and oral/gestural emission qualitatively; oral and written comprehension of words and sentences; repetition and reading aloud of words, pseudowords, and sentences; copy of a sentence; dictation of words, pseudoword, and sentence; oral naming of nouns and verbs; automatic speech of numbers and music; and nonverbal praxis. The same speech therapist conducted all assessments to ensure consistency.

The participant was also assessed with the Visual Naming and Recognition Test - TENOM^(15)^. This test evaluates oral naming of 90 black-and-white images and records immediate, delayed, and total naming accuracy, as well as linguistic and visual errors and unrecognized items. It was administered by a second speech therapist before the intervention, at the end of the intervention (10 sessions), and 30 days after the end of treatment. During the study period, the participant paused the speech therapy sessions provided by the hospital team.

### Study design

#### tDCS

The tDCS was applied with a constant current using two electrodes (5 x 7 cm) surrounded by saline-soaked sponges placed on the head and fixed with elastic tape. The placement of the electrodes was based on the universal 10-20 electrode array system.

The anode was placed in the left inferior frontal gyrus at point F5 and the cathode in the contralateral supra-orbital region at point Fp2. The subject received active stimulation of 2 mA for 20 minutes.

The two-week intervention included a two-day break between weeks. The two-week intervention consisted of daily 45-minute sessions of word-finding therapy combined with 20 minutes of tDCS stimulation at the beginning of the session.

The tDCS was applied by a speech therapy student under the supervision of a speech therapist trained in the use of this technique. The therapist administering the intervention was not blind to the results of the evaluations, as she had to have access to the evaluations to select the items to be used for the treatment.

The tDCS protocol used in this study was based on previous evidence demonstrating the benefits of anodal stimulation over the left inferior frontal gyrus (F5) for improving naming, repetition, and speech motor planning tasks in individuals with aphasia^(5)^. This cortical region is strongly associated with lexical access and verbal fluency—functions that were impaired in the present case of mixed aphasia combined with apraxia of speech. The cathodal electrode was placed over the contralateral supraorbital region (Fp2), following standard protocols in the literature^(7)^, with the aim of minimizing interference with other language-related areas.

### Behavioral language therapy

The therapy was performed by the same person who administered tDCS and was supervised by a speech therapist with more than 15 years of experience. It was combined with tDCS to improve lexical access and word retrieval, as most people with aphasia have difficulties with word retrieval.

All sessions were videotaped and reviewed by the researchers in this study, who recorded the accuracy of the productions.

### Stimulus selection and intervention

For the selection of stimuli and design of the speech therapy intervention, we followed the methodology of the study by Kendall et al.^(16)^, which utilized nouns spanning six distinct semantic categories. For this study, the following categories were adopted: Body Parts, Food, Emotions, Animals, Clothes, and Objects, represented by real photos from sold materials such as boxes of Super Duper and the Internet. The aim was to address the communicative needs of the participants involved in the study.

In the first session, ten items from each category were trained. In each session, new items were added incrementally. Eight new items were introduced in the second session; six in the third and fourth sessions; and four in the fifth. These were added to those previously trained. Items were selected based on their communicative relevance to the patient, regardless of semantic category.

For the second week of intervention, a new training set was selected, keeping only the stimuli that the patient had difficulty naming in the first week of intervention. Figures that the participant had difficulty naming during evaluation were selected for training. In addition, more complex terms with low lexical frequency were selected for the second week of intervention. In the follow-up, the stimuli that the patient had difficulty evoking were retrieved.

The first terms that the participant could not name correctly were selected, i.e. 45 words. These were divided into two groups according to word length and frequency: a group of 23 words used for therapy in the second week and a group of 22 control words used to study the effects of generalization to untrained terms.

The intensive word retrieval therapy was based on Hierarchical Cueing Therapy^(17)^, in which the participant received progressively structured cues to support correct word production. To this end, a structured sequence of cues was used, as follows: (1) “What is this?” (e.g., show a photo of a tree); (2) “Can you write the word?”; (3) graphic cues (e.g., the number of letters); (4) phonological cues (e.g., the first sound, /a/); (5) semantic associations (e.g., “Can you tell where you can find this?”); (6) the therapist says the word (“tree”); (7) repetition of the target word. The cues used during the training were personalized for the participant, always progressing from the most challenging to the easiest.

### Data analysis

A descriptive analysis was performed to characterize the data. Data were grouped into tables in which the quantitative results before and after the intervention were presented.

The results of the assessments conducted with the ABLA Battery are shown in Table 1 at six different time points: one week before the start of treatment (A1), after the first session (A2), after five sessions (A3), after 10 sessions (A4) and after 30 days without intervention (A5).

**Table 1:** Comparison of the results of the Brief Language and Aphasia Assessment Battery – ABLA

ABLA Tasks	A1	A2	A3	A4	A5
Directed Interview/18	18	18	18	18	18
Automatic speech (form)/4	3	3	2	3	4
Automatic speech (content)/4	2	4	2	3	2
Oral comprehension/11	6	7	6	7	7
Written comprehension/11	6	5	5	6	6
Copy/8	0	0	0	0	0
Writing to dictation/7	1	1	1	1	2
Repetition/12	7	5	7	7	9
Reading aloud/12	8	9	9	9	8
Nonverbal praxis/24	24	24	24	24	24
Oral naming/24	14	17	14	13	17

**Caption:** A1 = initial assessment, without tDCS; A2 = assessment after the first tDCS session; A3 = assessment after the fifth tDCS session; A4 = assessment after the tenth tDCS session; A5 = assessment after 30 days without tDCS

Table 1 shows that the participant consistently performed well on the directed interview and nonverbal praxis tasks from A1 onward. In the copy task, however, performance fell short of expectations as he reproduced the sentences mechanically across all assessments.

In the automatic speech task, it can be observed that the participant was successful in form A5, while in the other assessments, he showed formal paraphasias (e.g. four per quarter) and achieved a lower score. In terms of automatic speech content, he had difficulty singing the song "Happy Birthday", even with the possible facilitations according to the ABLA application manual.

In the oral and written comprehension tasks, difficulties were observed at the sentence level, especially with complex sentences. When writing to dictation, he only wrote the regular word and refrained from writing the others. In A5, however, he wrote the pseudoword correctly. In the repetition and pre-reading tasks, difficulties with pseudowords were observed in both tasks.

In addition, the participant could not repeat the phrase in the repetition task. In the naming task, the greatest difficulties were observed with verbs, in addition to the presence of paraphasias with nouns.

Chart 1 shows the qualitative analysis of linguistic errors committed by the participant during the ABLA tasks across the five moments.

**Chart 1:** Linguistic errors in the Brief Language and Aphasia Assessment Battery – ABLA

**Linguistic errors**	**A1**	**A2**	**A3**	**A4**	**A5**
Anomia	Yes	Yes	Yes	Yes	Yes
Semantic paraphasia	Yes	No	No	No	No
Formal paraphasia	Yes	Yes	Yes	No	No
Phonemic Paraphasia	Yes	Yes	Yes	Yes	Yes
Phonemic paraphasia/paralexia	No	Yes	Yes	Yes	Yes
Verbal paraphasia	No	Yes	Yes	Yes	Yes
Paragraphy	Yes	No	No	No	No
Jargon	No	No	Yes	No	No
Neologism	Yes	Yes	Yes	Yes	Yes
Perseveration	No	No	No	Yes	No
Regularization errors	Yes	No	No	No	No
Lexicalization errors	Yes	Yes	Yes	Yes	No

**Caption:** A1 = initial assessment, without tDCS; A2 = assessment after the first tDCS session; A3 = assessment after the fifth tDCS session; A4 = assessment after the tenth tDCS session; A5 = assessment after 30 days without tDCS

Chart 1 shows that since A1 many difficulties in speaking and writing have impeded the comprehensibility of the participant. As the treatment progressed, there were improvements in emission, with formal and semantic paraphasias and regularization errors decreasing. Anomia persisted, as did verbal, phonemic, and phonetic paraphasias and neologisms, but in a less obvious way when considering the tasks presented.

Table 2 shows the quantitative data from before the intervention, immediately after the intervention, and the evaluation after thirty days of TENOM.

**Table 2:** Comparison of the results of the naming and visual recognition test – TENOM

		A1		A4		A5
	score obtained/maximum score	Classification	score obtained/maximum score	Classification	score obtained/maximum score	Classification
immediate successes	26/90	Suggestive of moderate to severe deficit	56/90	Average score	51/90	Suggestive of mild deficit
slow successes	19/90	Suggestive of mild deficit	9/90	Average score	14/90	Average score
total successes	45/90	Suggestive of moderate to severe deficit	65/90	Average score	65/90	Average score
language errors	18/90	Suggestive of moderate to severe	8/90	Suggestive of moderate to severe deficit	7/90	Suggestive of mild deficit
visual errors	14/90	Average score	4/90	Average score	8/90	Average score
unrecognized item	13/90	Suggestive of moderate to severe deficit	13/90	Suggestive of moderate to severe deficit	10/90	Suggestive of moderate to severe deficit

**Caption:** A1 **=** assessment before the intervention; A4 = assessment immediately after the intervention; A5 = assessment after 30 days

Table 2 shows that the accuracy and efficiency of the participants have improved significantly since A4, with the number of correct answers increasing and errors decreasing. These results remained consistent even 30 days after the intervention ended.

**Figure 1:**
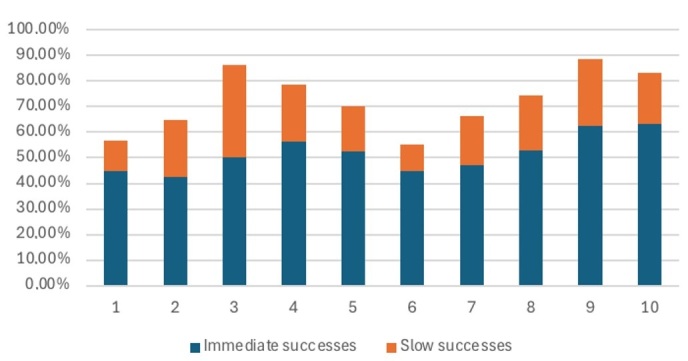
Quantitative data from the behavioral intervention

Figure 1 shows the quantitative data obtained in the behavioral intervention in each therapy session (10 sessions).

As shown in Figure 1, the participant achieved his best performance in the ninth session, and stabilized in the tenth session. The therapy significantly improved both naming accuracy and reaction time. The data shows that immediate correct responses rose steadily from the first to the fourth session, and declined during the fifth and sixth sessions, probably because of the time without treatment and the difficulty with the new set of stimuli.

Regarding the effects of tDCS on the generalization of unpracticed terms, at the last assessment (A5) with TENOM, the patient answered 10 out of the 22 selected terms accurately, resulting in a generalization percentage of 45.45%.

## DISCUSSION

The hypothesis was that tDCS, combined with intensive speech therapy, would enable the participant to improve or reduce the signs associated with aphasia. Thus, the results confirm the hypothesis and show that tDCS, in combination with intensive conventional speech therapy, enhances treatment outcomes and reduces the symptoms associated with aphasia. Our findings are consistent with those of Campanella et al.^(5)^, who found that tDCS in combination with speech therapy can be effective in patients with aphasia due to stroke.

Following the intervention, there were notable improvements in several areas, including spontaneous speech, oral comprehension, naming, and repetition. In a study where the injured left hemisphere received anodal stimulation, most patients showed significant improvement in various oral language tasks, such as describing pictures, naming nouns and verbs, repeating words, and reading^(18)^.

The improvement observed in tasks that were not directly targeted in therapy, such as oral comprehension, may be related to the overlap of neural networks involved in both naming and auditory comprehension, particularly within the superior temporal gyrus. Previous studies have shown that intensive training in one language function can lead to gains in related skills^(3,8,19)^.

As for the qualitative results of the ABLA Battery, the participant showed reductions in formal and semantic paraphasias, as well as in regularization errors. Meinzer et al.^(7)^ conducted a similar study and also obtained positive gains using speech therapy associated with tDCS by stimulating the same areas of interest as this study. The authors also demonstrated that tDCS associated with speech therapy allows the participant to improve or reduce the signs associated with aphasia. In the study, the authors demonstrated that the person with aphasia improved their performance in naming tasks throughout the training period.

The results also show that intensive word-finding therapy affected some language skills other than naming, such as oral comprehension. One possible explanation is that oral comprehension and naming abilities depend on neural networks with shared brain structures, such as the superior temporal gyrus. In this way, intensive naming training can promote both types of processes. Moreover, progress is maintained over an extended period, even without intervention (A5, Table 1).

Other studies that conducted an intervention with tDCS in combination with intensive word retrieval therapy achieved positive results in the naming task^(20)^. In addition, tDCS has been shown to help with immediate recall of everyday items/objects and supports generalization to untrained items. Similar findings were noted in other studies, which additionally reported generalization to untrained items in single-case reports^(19)^.

Despite the short time interval between the application of the tests, improvements were sustained after 30 days of intervention, which may indicate a therapeutic effect. In addition to the improvements observed in the tests, qualitative analysis revealed a reduction in paraphasias. Moreover, the participant showed a significant percentage of correct responses for non-practiced words, which may indicate gains that were not restricted to the specific treatment.

The literature indicates that tDCS may enhance the consolidation of therapeutic gains, even after the end of the intervention period^(19,20)^. The present study supports these findings, demonstrating maintenance of performance in both trained tasks and untrained items 30 days after the intervention was completed.

TENOM also assessed reaction time. Although immediate adaptations were still deficient, the severity of the deficit decreased. On the other hand, both slow and total correct responses showed improvement. Other studies have also documented enhanced naming accuracy and speed^(18)^. It is worth pointing out the relationship between reaction time and aging, as the latter may be associated with a decline in cognitive abilities in older people. Reaction time is an inherent aspect of aging and not unique to aphasia^(21)^. Processing speed mediates several age-related functions, including functions involving fluency and crystallization skills, such as phonological fluency and naming.

Despite the factors mentioned, we have shown that significant improvements are possible even in elderly individuals with low educational backgrounds. This should be taken into account both in clinical practice and public health, which often do not invest in the communication of these individuals. In addition, people with aphasia tend to be older and already have some age-related language decline, which a stroke may further worsen. Therefore, there is a need to invest in this population, as intensive treatments, both alone and in combination with tDCS, can lead to meaningful gains.

A limitation of this study is that the results cannot be generalized to the population, as they are limited to the subject studied. Furthermore, it is crucial to highlight that although the study provides a broad overview of the results, future research should explore this topic further, including the importance of a control group. Future studies should also include follow-up sessions over an extended period to assess how long the effects persist.

## FINAL COMMENTS

After the therapeutic intervention, naming performance improved for both trained and untrained words, accompanied by enhancements in listening comprehension. In addition, a decrease in linguistic errors, such as formal and semantic paraphasias and regularization errors, was observed in a long-lived person with low education.

These results show that the treatment was effective in this single-case study. Thus, this study confirms the data found in the literature on the benefits of a combination of tDCS and intensive word retrieval training under subacute conditions. To obtain more conclusive data, researchers should conduct randomized controlled clinical trials.
